# Optimization of treatment strategy by using a machine learning model to predict survival time of patients with malignant glioma after radiotherapy

**DOI:** 10.1093/jrr/rrz066

**Published:** 2019-10-28

**Authors:** Takuya Mizutani, Taiki Magome, Hiroshi Igaki, Akihiro Haga, Kanabu Nawa, Noriyasu Sekiya, Keiichi Nakagawa

**Affiliations:** 1 Graduate Division of Health Sciences, Komazawa University, Tokyo, Japan; 2 Department of Radiation Oncology, National Cancer Center Hospital, Tokyo, Japan; 3 Graduate School of Biomedical Sciences, Tokushima University, Tokushima, Japan; 4 Department of Radiology, The University of Tokyo Hospital, Tokyo, Japan

**Keywords:** malignant glioma, support vector machine, survival time prediction, dose–volume histogram features, clinical features

## Abstract

The purpose of this study was to predict the survival time of patients with malignant glioma after radiotherapy with high accuracy by considering additional clinical factors and optimize the prescription dose and treatment duration for individual patient by using a machine learning model. A total of 35 patients with malignant glioma were included in this study. The candidate features included 12 clinical features and 192 dose–volume histogram (DVH) features. The appropriate input features and parameters of the support vector machine (SVM) were selected using the genetic algorithm based on Akaike’s information criterion, i.e. clinical, DVH, and both clinical and DVH features. The prediction accuracy of the SVM models was evaluated through a leave-one-out cross-validation test with residual error, which was defined as the absolute difference between the actual and predicted survival times after radiotherapy. Moreover, the influences of various values of prescription dose and treatment duration on the predicted survival time were evaluated. The prediction accuracy was significantly improved with the combined use of clinical and DVH features compared with the separate use of both features (*P* < 0.01, Wilcoxon signed rank test). Mean ± standard deviation of the leave-one-out cross-validation using the combined clinical and DVH features, only clinical features and only DVH features were 104.7 ± 96.5, 144.2 ± 126.1 and 204.5 ± 186.0 days, respectively. The prediction accuracy could be improved with the combination of clinical and DVH features, and our results show the potential to optimize the treatment strategy for individual patients based on a machine learning model.

## INTRODUCTION

As prognostic prediction models have been used to evaluate radiation treatment plans, tumor control probability (TCP) [1] and normal tissue complication probability (NTCP) [2], the optimization of the treatment plan through these models is called biological optimization (BIOP) [3]. By considering these biological models, dose distribution could be improved [3–5]. However, these biological models only consider the radiation dose information obtained from a dose–volume histogram (DVH). There is a probability that not only the information obtained from DVH but also clinical features about patients might affect the prognosis of patients. In fact, Curran *et al*. [6] reported that the survival time after radiotherapy of patients with glioma, who are >50 years old and have an abnormal mental status, is short. Therefore, a prognostic prediction model could possibly be constructed with high accuracy by considering clinical features in addition to the detailed DVH features. In this study, a prognostic prediction model was constructed using the combination of clinical features and detailed DVH features, and the model was used to demonstrate the optimization of treatment strategies for individual patients.

To predict prognosis after radiotherapy by simultaneously considering the clinical and DVH features, a machine learning model was used. In recent years, many researchers have applied various machine learning models in the field of radiotherapy, and shown their usefulness [7–9]. Some studies have shown that support vector machines (SVMs) achieve superior performance over other statistical and machine learning methods [7,10,11], and therefore the SVM model was used in this study. If the machine learning model is constructed with high accuracy, the correspondence between input features and output is clear.

If the survival time is set as the output of a machine learning model, input features related to treatment strategy can be directly controlled to maximize the survival time. Thus, the possibility exists that prognosis can be improved by using machine learning. Although many studies using machine learning models for the prediction of patient outcome have been reported, to the best of our knowledge, no studies in which a prediction model has been used for optimization of treatment strategies for patients with malignant glioma have been reported. Therefore, in this study, the change of the predicted survival time after radiotherapy was evaluated by changing some input features. The purpose of this study was to predict the survival time of patients with malignant glioma after radiotherapy by combining the clinical and DVH features by using an SVM model. In addition, the relationship between the predicted survival time and some input features was investigated to assist decision making in the treatment strategy.

## METHODS AND MATERIALS

First, three feature groups (clinical features, DVH features and their combination) were prepared. By using these feature groups, appropriate input features and parameters were selected for SVM models according to a genetic algorithm (GA), and the prediction accuracies of the three models were compared. Finally, the change of the predicted survival time with the change in some input features was investigated using the SVM model constructed with the combination of clinical and DVH features.

### Patients and input candidate features

The details of 35 patients with malignant glioma (oligodendroglioma: 2, anaplastic astrocytoma: 3, glioblastoma: 30), who were treated at the University of Tokyo Hospital between April 2006 and March 2013, were used in this study; these details were tracked until the patients’ deaths. This study was approved by the University Institutional Review Board. The patient characteristics are shown in Table 1. All patients underwent gross-total or subtotal resection or biopsy, followed by adjuvant chemoradiotherapy with prescription doses of 30–80 Gy. A total of 204 candidate features that included 8 clinical features and 196 DVH features were prepared (Table 2). The clinical target volume (CTV)_extend_ is defined as the surrounding edema with a 1.5–2-cm margin, while the planning target volume (PTV)_extend_ is defined as CTV along with a 5-mm margin for setup error. CTV_local_ is defined as the residual tumor + tumor bed along with 1.5–2-cm margin or the edema surrounding the tumor (smaller than CTV_extend_). PTV_local_ is defined as CTV_local_ along with a 5-mm margin for setup error. *D_X_* represents the dose irradiated to a volume of *X* cm^3^ (or %) or more, and *X* = 98, 95–50 (five intervals) and 2. Further, *V_X_* represents the volume of the irradiated dose of *X* Gy or more, where *X* = 80–5 (five intervals).

**Table 1:** Patient characteristics

	Glioma patients (*n* = 35)
Age	
Median (range), years	64 (11–92)
Sex	
Women	12
Men	23
Survival time	
Median (range), days	504 (64–1279)
Mental status	
Normal	19
Abnormal	16
Tumor location	
Frontal lobe	17
Temporal lobe	10
Parietal lobe	1
Other	7
Symptom duration	
Median (range), days	93 (27–3119)
Prescription dose	
Median (range), Gy	60 (30–80)
Treatment duration	
Median (range), days	50 (19–80)

**Table 2:** Candidate input features for the prediction of survival time after radiotherapy

Clinical features (8)	DVH features (196)
Age	Gammaknife
Gender	Target volume
Histology	Prescription dose
Mental status	Treatment duration
Chemotherapy	Biological effective dose (BED)
Tumor location	PTV_local_, *D_x_*, *V_x_*, max, min, mean
Surgical resection	CTV_local_, *D_x_*, *V_x_*, max, min, mean
Symptom duration	PTV_extend_, *D_x_*, *V_x_*, max, min, mean
	CTV_extend_, *D_x_*, *V_x_*, max, min, mean
	*D_x_*: *D*_98_, *D*_95_–*D*_50_ (five interval), *D*_2_
	*V_x_*: *V*_80_–*V*_5_ (five interval)

### SVM-based survival time prediction

An SVM is mostly used for classification but can be modified for a regression problem [12]. In this study, the survival time of patients with glioma after radiotherapy was predicted as a regression problem. The SVM maps the training data into a high-dimensional feature space through a kernel function. As the radial basis function (RBF) kernel is generally the most commonly used kernel function because of its better generalization ability [13], it was used in this study.

The SVM model as a regression model must select three parameters: *C*, *ε* and *γ* [12]. Parameter *C* is a regularization parameter that controls the tradeoff between training error and model complexity. Parameter *ε* controls the width of the insensitive zone that allows the error and parameter *γ* controls the amplitude of the RBF kernel and the generalizability of the SVM.

### Selection of input features and parameters for the SVM

**Fig. 1:**
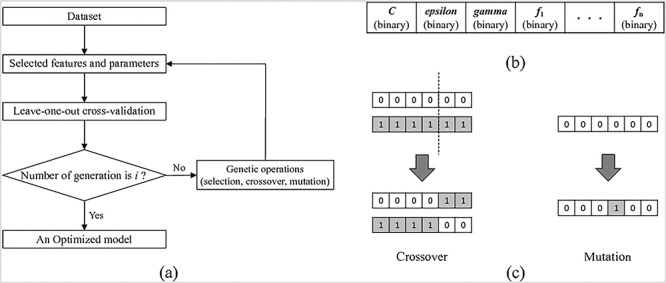
(a) Flowchart of feature selection and parameter optimization by using a genetic algorithm. (b) Encoding of the chromosome comprising four parts: *C*, ε, γ, and the features mask. (c) Genetic crossover and mutation operation.

Figure 1(a) shows the flowchart for the selection of input features and parameters by using the GA. The combination of input features affects the selection of appropriate parameters of the SVM, and vice versa. Therefore, these two problems should be addressed simultaneously. As the GA has been recently used for the simultaneous selection of SVM features and parameters [14,15], it was considered in the current study for simultaneously selecting input features and parameters for the construction of the SVM model. The input features and parameters are expressed in the GA with chromosomes; this is one such solution to the given problem. In fact, the GA works with a subset of all possible solutions called “population.” The optimal solution is obtained through a series of iterative calculations (generations). The detailed process of the GA-based selection of input features and parameters is as follows.

First, each “individual” (one set of input features and parameters) in the population should be initialized by a chromosome [14]. To represent parameters of the SVM and input features with a chromosome, a binary encoding technique [14,15] was used. In binary encoding, parameters of the SVM (*C*, ε and γ) are expressed in their binary form. That is, in the case of feature selection, the bit value of “1” implies that the feature is selected, whereas “0” implies that the feature is not selected (Figure 1(b)). Then, a fitness function that represents the suitability of each individual was calculated, and genetic operations (selection, crossover and mutation) were applied [14,15] (Figure 1(c)). The calculation of the fitness function and genetic operation were repeated until termination was achieved.

The fitness function was used for the evaluation and comparison of each candidate individual. In this study, Akaike’s information criterion (AIC) [16] for the SVM was used as the fitness function, and smaller fitness values indicated that an individual was fit. A simple model with a small number of features is more likely to be selected using AIC.

For AIC calculation, the SVM model was trained and validated through leave-one-out cross-validation (LOOCV) that maintained the best compromise between computational cost and reliable estimates [17]. LOOCV involves the use of one observation as the validation set and the remaining observation as the training set. This procedure was repeated until every observation was used once.

The iterative selection process of the GA was terminated when the evolution reached 1000 generations. The other parameters for the GA were as follows: population size was 500, crossover rate was 0.8 and mutation rate was 0.3. These parameters were referenced from relevant literature [14,15].

### Prediction-accuracy validation of the SVM model

The survival time of patients with glioma was predicted using the SVM model through LOOCV, and the absolute difference between the actual and predicted survival times (residual error) was calculated. The mean residual error of all patients was used as the performance evaluation index.

### Investigation of influence of input features on predicted survival time

By using the SVM model, we investigated how the predicted survival time changed with the change in the values of some input features selected through the GA. In this study, the SVM model constructed with the combination of clinical and DVH data was used as the prediction model, and the “prescription dose” and “treatment duration” were used in this investigation. We predicted the survival time by changing “prescription dose” in three steps. First, the prescription dose was changed in the simulation. Second, the DVH parameters selected by the GA were changed corresponding to the prescription dose in the simulation. Finally, by using the changed DVH parameters as input features, the survival time was predicted. Wilcoxon signed rank test was applied as a non-parametric statistical test. All statistical analyses were performed using R version 3.1.4.

## RESULTS

### Prediction accuracy of each prediction model

**Fig. 2:**
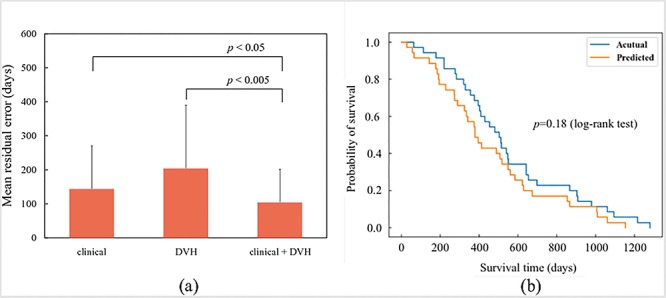
(**a**) Mean residual error from LOOCV by using each feature group, i.e. clinical features, DVH features and combination of clinical and DVH features. The prediction accuracy when using the combination of clinical and DVH features was significantly improved (*P* < 0.05 and 0.005, respectively, paired *t*-test). (**b**) Survival curve using actual and predicted survival time. There is no significant difference (*P* = 0.18, log-rank test).

Figure 2(a) shows the mean residual errors of each prediction model. Mean ± standard deviation of LOOCV when using the clinical features, DVH features, and the combination of these features was 144.2 ± 126.1, 204.5 ± 186.0 and 104.7 ± 96.5 days, respectively. The model constructed using the combination of clinical and DVH data produced significantly better performance than those constructed using clinical data (*P* < 0.01, Wilcoxon signed rank test) and DVH data (*P* < 0.01, Wilcoxon signed rank test). The features selected from each feature group according to the GA are shown in Table S1 (see online supplementary material), and the distribution of each residual error using the combination of clinical and DVH features, clinical features only, and DVH features only is shown in Figure S1 (see online supplementary material).

The GA was used for the simultaneous and automatic selection of input features and parameters. To show that the GA can select input features and parameters appropriately, the convergence of fitness values was investigated, and the performance of the GA was compared with that of the grid-search algorithm [18] for the same range of search parameters. Details of the comparison of the GA and grid search are shown in Figure S2 (see online supplementary material).

Figure 2(b) shows the Kaplan–Meier survival curves of the actual and predicted survival times, with no significant difference between the curves (*P* = 0.18, log-rank test). The SVM model constructed by combining the clinical and DVH features was used to generate the Kaplan–Meier survival curves.

### Relationship among prescription dose, treatment duration and predicted survival time

**Fig. 3:**
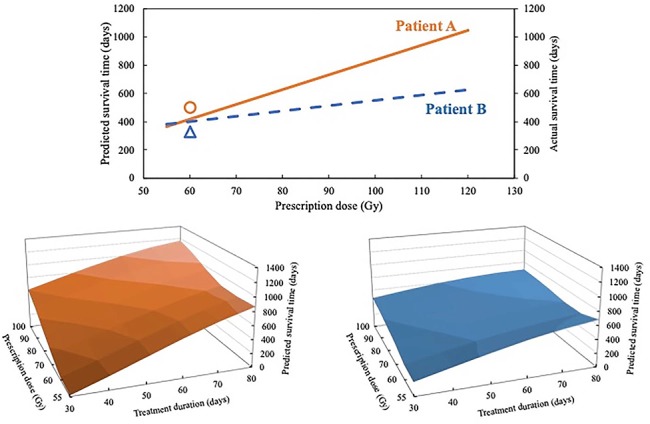
Result of investigating the change in the predicted survival time when changing prescription dose and treatment duration as input features. Top: relationship between the prescription dose and predicted survival time of each patient. The round (○) symbol shows the actual survival time and prescription dose of patient A, and the triangle (△) symbol shows the actual survival time and prescription dose of patient B. Bottom: the result of adding treatment duration of each patient.

Figure 3 (top) shows the relationship between the prescription dose and predicted survival time for two patients. The effect of dose escalation differed according to each patient. Figure 3 (bottom) shows the result of adding the treatment duration of each patient. The influence of prescription dose and treatment duration on the survival time after radiotherapy was different for each patient.

## DISCUSSION

In this study, the clinical and DVH features were combined for constructing a prognostic prediction model. By considering more input features related to prognosis by using a machine learning model, the prediction accuracy was improved. Moreover, we showed the potential to optimize the treatment strategy for an individual patient by using the prediction model. By using our methods, there is a possibility that prescription dose and treatment duration can be controlled to improve survival time of individual patients after radiotherapy.

The prediction accuracy of the SVM model constructed using the combination of clinical and DVH features was significantly improved compared with the accuracies of those models constructed using only clinical features and only DVH features. This result indicates that the clinical features influence the prognosis of glioma patients as reported by Curran *et al*. [6]. Therefore, prognostic prediction models are recommended to be developed by considering not only radiation dose information from DVH but also clinical features.

The survival time of patients with glioma was predicted as a regression problem in this study. Some reports predicted the survival time for patients with glioma as a classification problem [6,19,20]. However, to the best of the authors’ knowledge, no study has reported this problem as a regression problem. For comparison with other reports, the survival time was binarized with the median value and the prognosis of glioma patients was predicted as a classification problem. The area under the curve (AUC) of our SVM model was 0.91, which is equivalent to or marginally higher than the values in the past reports (AUC = 0.7–0.9) [21,22]. Chaddad *et al*. [21] achieved a prediction performance of AUC = 0.85 using radiomic features in presurgical magnetic resonance (MR) images. Papp *et al*. [22] reported AUC = 0.9 based on a model with radiomic features calculated using LS-methyl-11C-methionine (11C-MET) positron emission tomography (PET) images. In the classification problem, the patients were classified into two groups according to their survival time (for example, class 1: survival time >50 days, class 2: survival time ≤50 days). Therefore, the treatment strategy was optimized with respect to each patient group instead of individual patients. However, in regression, as in this study, the survival time is predicted as a continuous value. As different survival times are predicted for each patient, the treatment strategy can be optimized for individual patients.

Conventionally, the quality of radiotherapy is judged according to the physical quantities such as DVH parameters. However, optimization of radiotherapy with respect to these parameters is an indirect method because the optimization is based on the assumption that these parameters correlate with the prognosis after radiotherapy. Therefore, we investigated the direct relationship between some input features and the predicted survival time after radiotherapy by using a machine learning model. In this study, the input features of prescription dose and treatment duration were used for optimization because they are continuous and related to treatment strategy. The motivation for this investigation was to personalize radiotherapy for individual patients. If prognostic prediction models are constructed with high accuracy based on this study, it is possible to control some input features related to the treatment strategy to improve the predicted survival time after radiotherapy, leading to the realization of precision medicine in the field of radiotherapy. As shown in Figure 3, the effect of dose escalation and extension of treatment duration differed according to each patient. Therefore, there is a probability that the optimal prescription dose and treatment duration vary with respect to each patient. This investigation could be the first step for the optimization of the prescription dose and treatment duration for individual patients.

In this study, an SVM model was used as a machine learning model to predict the survival time of patients with glioma after radiotherapy because of its superior performance. In recent years, multilayer neural networks called “deep learning networks” have achieved higher prediction accuracy than other machine learning models; however, deep learning methods require a huge amount of data to achieve high prediction accuracy [23,24]. Although You *et al*. [25] proposed a hybrid model based on convolutional neural network (CNN) and SVM, a large dataset is still needed. In the case of a small dataset, simpler machine learning models, such as SVM and multiple linear regression [26] (MLR), could perform with high prediction accuracy [7]. As an additional study, the performances of the SVM and MLR models were compared, as shown in Figs S3 and S4. As shown in Fig. S4(a), the SVM model had higher generalizability to the separated dataset than the MLR model. Although random forests have also been used for small datasets, the random forest is generally used as a classifier for classification problems [27,28]. In this study, survival time after radiotherapy was predicted as a regression problem. Therefore, we compared the SVM and MLR models in this study.

Future studies are expected to increasingly report about the use of machine learning models in not only prognostic prediction [29,30] but also optimization of treatment strategies in the field of radiotherapy. Therefore, clinical trials to ensure the usefulness of optimizing treatment strategies using machine learning models will become necessary, e.g. the use of prospective tests to ascertain whether radiotherapy optimized through a machine learning model significantly improves prognosis. If such clinical trials are performed in many facilities in the future, the usefulness of the treatment-strategy optimization by using machine learning models will be ensured, and machine learning methods will be incorporated into actual radiotherapy.

The current study has some limitations. First, the sample size of our dataset is 35 because only the cases that could be tracked until death were used. Second, adversarial events were not considered. For example, if we tried to optimize the prescription dose and treatment duration, adverse events accompanying them must also be considered; however, our dataset does not contain features about adverse events because all patients died before the occurrence of any adverse events. Therefore, more samples and input features should be collected in future works. Radiomics features could be effective as input features to improve the prediction accuracy of survival time of patients with glioma. In recent years, the field of radiomics has attracted attention, and many studies have been published on this topic [31–33]. Radiomics refers to the comprehensive quantification of tumor phenotypes by applying a large number of quantitative image features [34]. In fact, Grossmann *et al*. [35] reported that radiomics provides prognostic value for survival and progression in patients with recurrent glioblastoma. In this study, however, radiomics information, such as presurgical MR images, were not obtained for survival time prediction because modalities and imaging sequences of the glioma image were not unified. To improve prediction accuracy using radiomics, modalities and imaging sequences should be unified [36,37]. Moreover, there are many other factors, that could not be obtained in this study, associated with a glioma patient’s prognosis [38], such as Karnofsky performance score (KPS), resection percentage and genomics features. The prediction accuracy may be increased by including these features.

## CONCLUSIONS

In conclusion, our result showed the potential to customize the treatment strategy for individual patients based on a machine learning model. Furthermore, the prediction accuracy could be improved by combining clinical and DVH features. To apply the prediction model to clinical use, a more absolute prediction accuracy and a prospective test are needed.

## Supplementary Material

04_Supplement_annotation_v6_CLEAN_rrz66
