# Supplementary material for: Optimization of treatment strategy by using a machine learning model to predict survival time of patients with malignant glioma after radiotherapy
Source: J Radiat Res. 2019 Oct 28;60(6):818–24. doi: 10.1093/jrr/rrz066 (PMC7357235; doi:10.1093/jrr/rrz066)
Supplement: 04_Supplement_annotation_v6_CLEAN_rrz66 [file 04_supplement_annotation_v6_clean_rrz66.docx]

**Supplemental data**

**Optimized input features of the SVM model by using the GA**

Table S1 shows the optimized input features of the SVM for each feature group, i.e., clinical features, DVH features, and the combination of clinical and DVH features, by using the GA.

**Table S1: Optimized input features of the support vector machine (SVM) obtained from each feature group by using the genetic algorithm (GA). These features were used for construction of prediction models.**

| Clinical features | DVH features | Clinical and DVH features |
| --- | --- | --- |
| Gender | Gammaknife | Histology |
| Mental status | PTV_local_ *V*_55_ (cm^3^) | Mental status |
| Chemotherapy |  | Tumor location |
| Tumor location |  | Surgical resection |
| Surgical resection |  | Symptom duration (day) |
| Symptom duration (day) |  | Treatment duration (day) |
|  |  | CTV_extend_ *D*_98_ (Gy) |
|  |  | CTV_extend_ *V*_55_ (cm^3^) |
|  |  | PTV_extend_ *V*_60_ (cm^3^) |

The optimized input features differed according to the candidate feature groups, indicating that effective features for the prediction of survival time are not independent but depend on the combination of input features. In other words, input features affect each other.

**Histogram of the residual error using each feature group**

We plotted the histogram of the residual error using each feature group.


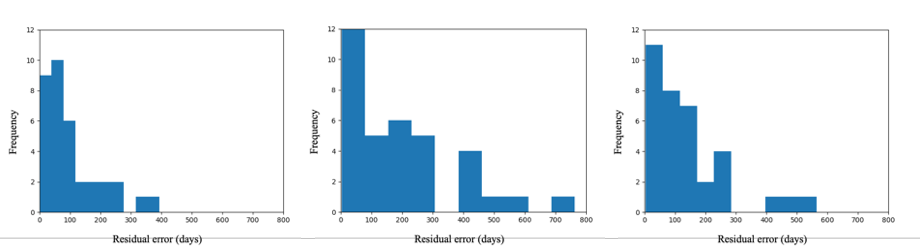


**Figure S1: (a) Histogram of residual error for clinical and DVH features. (b) Histogram of residual error for only clinical features. (c) Histogram of residual error for only DVH features.**

Figures S1(a), (b), and (c) are not normally distributed. In this case, t-test cannot be applied as a parametric statistical test. Therefore, Wilcoxon signed rank test was applied as a nonparametric statistical test.

**Convergence of fitness values and optimization of parameters of the support vector machine (SVM) model**

We checked the convergence of fitness value and validated the “optimizability” of the genetic algorithm (GA) by comparing with a grid search method.


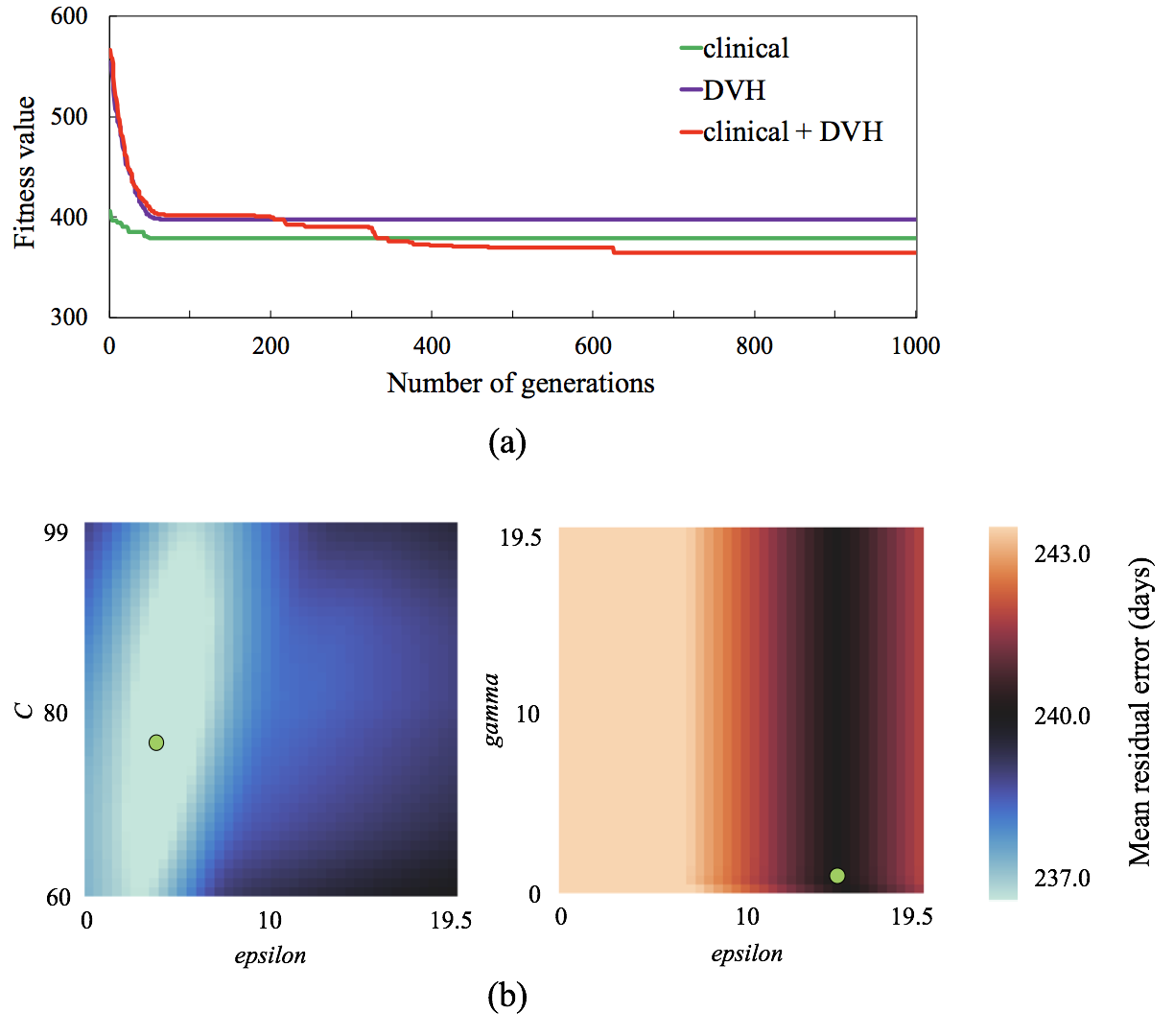


**Figure S2: (a) Relationship between fitness value and number of generations. (b) Heat maps of a grid search for optimal parameters of the support vector machine (SVM). (Green points show optimized parameters obtained through the genetic algorithm (GA).)**

Figure S2(a) shows the relationship between fitness values and number of generations. Until the fitness values reach a plateau, the number of generations varied depending on the feature group. Particularly, in the case of using the combination of clinical and DVH features, a large number of generations were required to reach plateau (approximately 600 generations). A total of 216 candidate features were included in the combination of clinical and DVH features. Therefore, the number of possible combination of input features increased, and a large number of generations were necessary to find optimal combinations of the input features.

Figure S2(b) shows the result of grid search for optimal parameters of the SVM, where the green points show optimized parameters obtained through the GA. Parameters of the SVM were optimized to the same values as those of the grid search by using the GA.

**Comparison of prediction errors of the SVM model and multiple linear regression (MLR) model optimized through GA**

The advantage of the SVM is its nonlinearity. In this section, the prediction errors of the optimized SVM model are compared with the optimized MLR model as a linear prediction model.


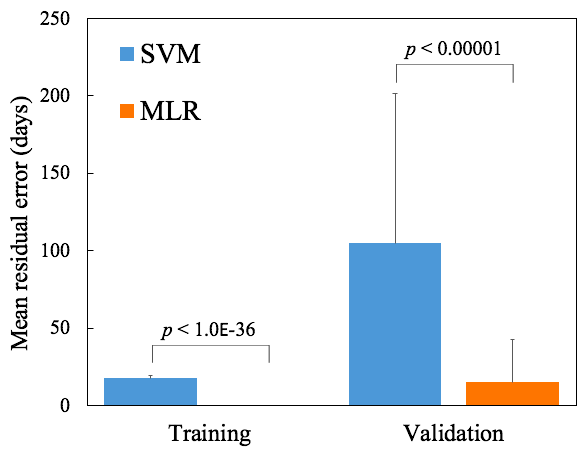


**Figure S3: Training and validation error of optimized support vector machine (SVM) and multiple linear regression (MLR) models.**

Figure S3 shows the prediction errors of the SVM model. The validation error of the MLR model was lower than that of the SVM model. There is a probability that the MLR model overfitted not only the training data but also the validation data because the training and validation data were used for optimization in our GA. Therefore, the generalizability of the prediction models must be evaluated using the “test data” not used for optimization.

**Evaluation for overfitting of the MLR and SVM models optimized by the GA**

One of the main issues with machine learning models, especially with small sample sizes, is overfitting to the training data. In this section, the generalizability of the optimized MLR and SVM models was evaluated using the test data. The optimized MLR and SVM were used as prediction models.


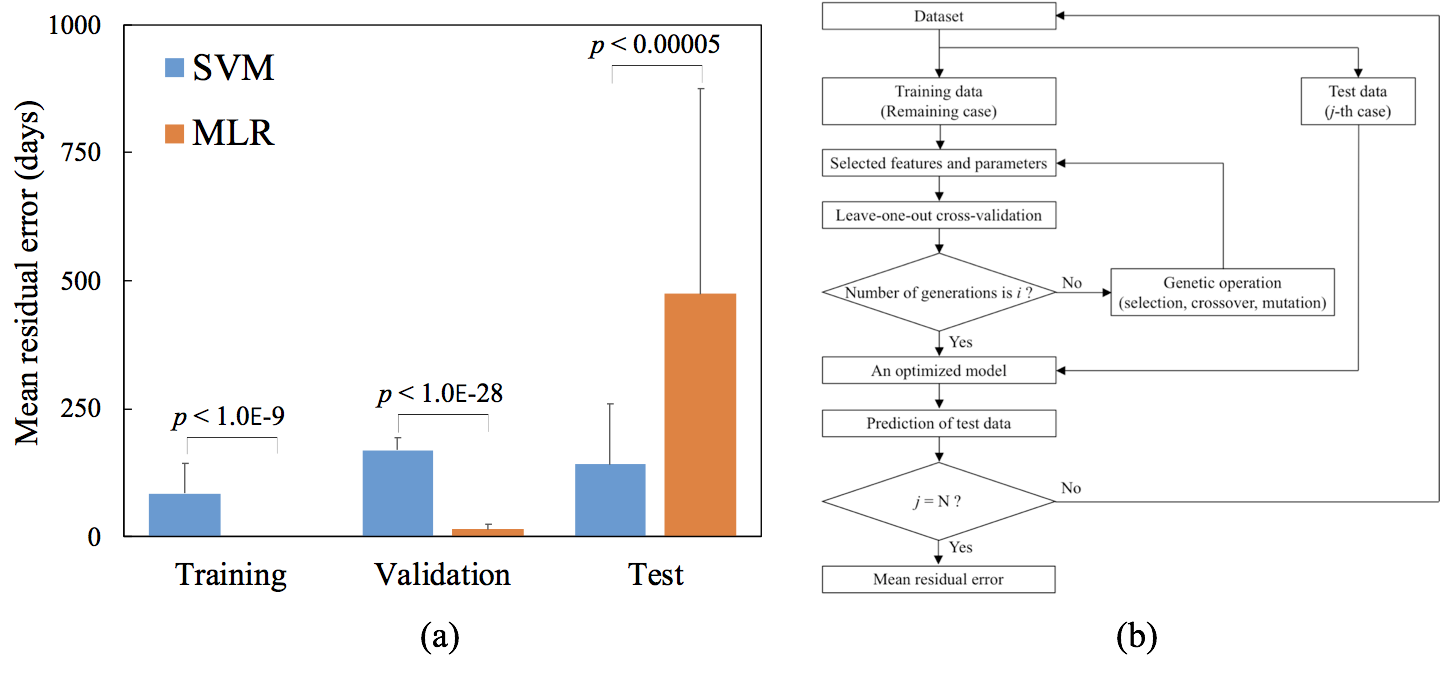


**Figure S4: (a) Training, validation, and test errors of the optimized support vector machine (SVM) and multiple linear regression (MLR) models. (b) Flowchart of optimization and evaluation of the SVM and the MLR models using the genetic algorithm (GA).**

Figure S4(a) shows the prediction errors of the optimized SVM and MLR models, and Figure S4(b) shows the flowchart for optimization and evaluation. The test error of the SVM model is significantly lower than that of the MLR model. Therefore, the SVM model has higher generalizability than the MLR model. The MLR model overfitted the data because the training and validation errors of the MLR model were very low. The SVM contains a regularization term in its cost function while the MLR does not. Thus, the SVM model has higher generalizability to the separated dataset.
